# Sexual behaviour and condom use of older adults living with HIV/AIDS in a treatment centre at Osogbo, south-west Nigeria

**DOI:** 10.4314/ahs.v24i4.20

**Published:** 2024-12

**Authors:** Samuel Anu Olowookere, David Ayobami Adewole, Omowonuola Olubukola Sonibare, Amos Akindele Ajayi, Ebenezer Gbenga Adepoju, Olumayowa Abimbola Oninla, Olubukunola Omobuwa, Emmanuel Oladayo Folami

**Affiliations:** 1 Obafemi Awolowo University, Community Health; 2 College of Medicine, University of Ibadan, Health Policy & Management; 3 Osun State University, Family medicine; 4 State Specialist Hospital, Preventive services; 5 Obafemi Awolowo University, Dermatology and Venereology; 6 Osun State University, Community Medicine; 7 Osun State University, Anesthesia

**Keywords:** Sexual behaviour, condom use, older adults

## Abstract

**Objective:**

This study assessed the condom use and sexual behaviour of older adults living with HIV/AIDS at Osogbo, Osun State, Nigeria.

**Methods:**

A descriptive cross-sectional study involving all adults receiving care at an HIV treatment centre that completed an interviewer administered questionnaire on their sexual behaviour and condom use. Data were analyzed using descriptive and inferential statistics.

**Results:**

A total of 186 older adults completed the study. Their mean age (SD) was 54.5 (5.6) years. There are 114 (61.3%) females and 72 (38.7%) males. Two fifth 87 (46.8%) were sexually active with over half using condom (45, 51.7%) at last sexual intercourse. The determinants of condom use at last sexual intercourse included age 50-59 years (OR=3.34, 95% CI=1.21-9.25, p=0.020), lower education (OR=3.00, 95%CI=1.04-8.69, p=0.043), being married or have a partner (OR=3.25, 95%CI=1.11-9.52, p=0.031), partner's awareness of respondents' HIV status (OR=13.00, 95%CI=4.25-39.80, p<0.0001) and stigma experience from partner (OR=11.70, 95%CI=4.03-33.99, p=0.0001).

**Conclusion:**

Older adults engage in high-risk sexual behaviour. It is necessary to encourage safer sex practices, stigma reduction and couple HIV counseling and testing.

## Introduction

HIV/AIDS is regarded worldwide as a disease most common among young people. However, statistics from several countries have proved that older people, that is people aged 50 years and above, are prone to the disease as much as the youth^1^-^3^. Studies have shown that this population are sexually exposed, with some older adults not protecting their sexual health^4^-^6^.

This change in sexual behaviour has been linked to the availability of drugs to treat sexual dysfunction which have extended the sexual life of older men^5^. This makes them prone to HIV infection due to low-risk perception of their vulnerability as they might not be as informed as their younger counterparts who have better access to information on HIV transmission and prevention^7^-^10^. Also, the majority of the present older generation became sexually active before the advent of HIV/AIDS hence did not grow up with information on the need to use a condom during sexual intercourse thus having a preference for unprotected sex^11^-^13^. Furthermore, older adults believed that younger people are more at risk of HIV than older people hence, are much less likely to adopt safer sex strategies such as using condoms during sex or getting tested for HIV^13^-^16^.

HIV is gradually becoming a disease of older adults as reports show that HIV prevalence is increasing dramatically among this population. The report of inconsistent condom use despite increasing sexual activity has been seen as one of the factors responsible for the HIV prevalence^12^-^14^. At-risk sexual behaviour and sexual activity becomes a common source of new Sexually Transmitted Infections including HIV infection^15^-^19^.

The success of highly active antiretroviral therapy has changed HIV/AIDS from a deadly disease or a chronic disease with young people earlier infected now living longer into old age. Therefore, declining HIV deaths lead to long time survivors aging with HIV that increases the proportion of people living with HIV in the general population^14^,^15^. The older adults tend to progress to AIDS faster than the young people living with HIV and have more co-morbid health conditions such as high blood pressure, high cholesterol, heart attack, angina, stroke, cancer, arthritis, asthma or diabetes^9^,^13^,^17^. This practice of unsafe sex after HIV diagnosis could lead to HIV transmission to unsuspecting partners who were not aware of their partners' HIV status. Several factors associated with sexual behaviour among older adults include inadequate HIV knowledge and prevention, inconsistent condom use in every sexual encounter, and multiple sexual partners.^18^-^20^

Since, the older adult is seen as ‘sexless’, the sexual and reproductive health of older people living with HIV/AIDS is largely neglected with no clear policy in several countries in sub-Saharan Africa including Nigeria^18^-^20^. This is because the focus has been on women in the reproductive age group who were assumed to be at-risk of HIV infection due to desire for pregnancy and sexual satisfaction^21^,^22^.

The present study assessed the sexual behaviour and condom use of older adults living with HIV/AIDS at HIV treatment centre, State Specialist Hospital, Osogbo, Nigeria.

## Methods

The HIV treatment centre, State Specialist Hospital, Osogbo was established by the Osun State Government in collaboration with the Global HIV/AIDS Initiative, Nigeria. It provides free comprehensive HIV/AIDS care and prevention which include HIV counseling and testing, prevention of mother to child transmission of HIV, provision of combination antiretroviral therapy to eligible HIV positive patients, and receive referral of HIV positive clients from all health care facilities in Osun State and beyond. The centre provides safer sex counselling sessions to all patients while male condoms are offered to sexually active patients. Also, the centre in collaboration with Osun State Agency for the Control of AIDS conducts outreaches where HIV counselling and testing are done by trained health workers with safer sex counselling sessions held during which male condoms are distributed freely to the participating public. The centre opens daily from 8:00 to 16:00 while emergency services are provided on 24 hour basis. It provides highly active antiretroviral therapy to eligible clients living with HIV/AIDS every Monday and Wednesday. The centre is headed by the ART coordinator with the support of other health care workers^9^,^13^.

This study employed a descriptive cross-sectional design. The study population included all eligible HIV positive persons aged 50 years and above receiving care at the clinic. Clients who were too ill to be interviewed and non-consenting clients were excluded from the study.

One hundred and eighty-six HIV positive older adults were enrolled into the study after sample size calculation using an appropriate statistical formula for descriptive health studies (n=Z2pq/d2) with 13.3% of sexually active older adults using condom consistently and non-response/attrition rate taken into consideration^23^,^24^.

The hospital records were obtained for all older adults on antiretroviral therapy between January and February 2020. Names and contact details were extracted from the hospital records and used to contact them. All contacted persons were approached to participate in the study and appointments were made for interviews at their next clinic appointments. The participants that did not come on their scheduled appointment day were contacted and if not available during the study period were replaced with the next participant on the list generated from the hospital records. They were subsequently interviewed in a predetermined, convenient place within the hospital till the calculated sample size was reached.

A semi-structured, pre-tested, interviewer-administered questionnaire which were derived from review of relevant literature was applied to the respondents. It elicited information on socio-demographic characteristics, sexual behaviour and condom use. These instruments were translated to Yoruba language and back translated to English language for Yoruba speaking respondents to ensure validity. Face validity of the instruments were done by the authors to ensure that the questions asked answered set objectives. The instruments were then pretested with ambiguous questions rephrased or removed. The interview was conducted individually in a private room designated for the research.

Ethical approval for the study was obtained from the State Hospitals Ethics and Research Committee with protocol number SHO/ERC/07/00025. A written informed consent was obtained from each respondent before administering the questionnaire. Confidentiality of collected data was maintained throughout the study as only the investigators stored data generated from the research.

The data were entered and analyzed using SPSS version 20. Simple descriptive and inferential statistics were performed and the results presented in tables. A bivariate logistic regression model was used to examine the relationship between condom use at last sexual intercourse (main outcome variable) and socio-demographic variables, partners' awareness of respondents' HIV status and stigma experience (explanatory variables). Results were expressed as odds ratios (ORs) and 95% confidence intervals (CIs). Tests were considered significant for a p-value at <0.05.

## Results

A total of 186 older adults completed the study. Their mean age (SD) was 54.5 (5.6) years ranging from 50 to 78 years. There are 114 (61.3%) females and 72 (38.7%) males. Most respondents were married/have a partner 164 (88.1%), had primary education 75 (40.3%), were trading 91 (48.9%) and lived in rural areas 110 (59.1%). Most respondents 90 (48.4%) stated that their partners were aware of their HIV status with majority 54 (60%) experiencing stigma. All respondents engaged in heterosexual vaginal sex with over two-fifth 87 (46.8%) sexually active in the previous three months. Condom use at last sexual intercourse was over half 45 (51.7%) among the sexually active respondents. (Table 1).

**Table 1:** Baseline socio-characteristics of the study population

Variable	Frequency	%
Age group (years)		
50-59	135	72.6
≥60	51	27.4
Sex		
Male	72	38.7
Female	114	61.3
Level of education		
None	13	7.1
Primary	75	40.3
Secondary	73	39.2
Tertiary	25	13.4
Marital status		
Married/have a partner	164	88.1
Widow/widower	12	6.5
Divorced	10	5.4
Occupation		
Unemployed	28	15.1
Trading	91	48.9
Artisan	44	23.7
Civil servant	23	12.3
Ethnicity		
Yoruba	163	87.6
Others	23	12.4
Religion		
Christianity	117	62.9
Islam	59	39.7
Traditional	10	5.4
Residence		
Urban	76	40.9
Rural	110	59.1
Sexual orientation		
Heterosexual	186	100
Sexually active		
Yes	87	46.8
No	99	53.2
Condom use at last sexual intercourse (n=87)		
Yes	45	51.7
No	42	48.3
Partner aware of HIV status		
Yes	90	48.4
No	96	51.6
Stigma experience from partner (n=90)		
Yes	54	60.0
No	36	40.0

Others= Igbo, Hausa, Edo, Itsekiri, Awori

Table 2 reports association between respondents' socio-demographic characteristics; HIV status disclosure, stigma experience and condom use at last sexual intercourse. Significant factors associated with condom use at last sexual intercourse included age 50-59 years, being married/ have a partner, having lower education, being unemployed, partner's awareness of respondents' HIV status and those experiencing stigma.

**Table 2:** Association between respondents' sociodemographic characteristics, HIV status disclosure, stigma experience and condom use at last sexual intercourse

Variable	Condom use at last sexual intercourse		Total	Test statistic
	Yes (%)	No (%)		
Age group (years)				
50-59	38 (59.3)	26 (40.7)	64	5.675; 0.017*
≥60	7 (30.4)	16 (69.6)	23	
Marital status				
Married/have a partner	39 (58.2)	28 (41.8)	67	4.908;0.027*
Not married	6 (30)	14 (70)	20	
Level of education				
None/primary	15 (71.4)	6 (28.6)	21	4.304; 0.038*
Secondary/tertiary	30 (45.5)	36 (54.5)	66	
Occupation				
Unemployed	9 (64.3)	5 (35.7)	14	1.054; 0.305*
Employed	36 (49.3)	37 (50.7)	73	
Religion				
Christianity	16 (36.4)	28 (63.6)	44	0.015**
Islam	24 (68.6)	11 (31.4)	35	
Traditional	5 (62.5)	3 (37.5)	8	
Ethnicity				
Yoruba	29 (43.9)	37 (56.1)	66	6.636; 0.010*
Others	16 (76.2)	5 (23.8)	21	
Residence				
Urban	16 (44.4)	20 (55.6)	36	1.303; 0.254*
Rural	29 (56.9)	22 (43.1)	51	
Partner aware of HIV status				
Yes	40 (71.4)	16 (28.6)	56	24.437; 0.0001*
No	5 (16.1)	26 (83.9)	31	
Stigma experience from partner				
Yes	39 (72.2)	15 (27.8)	54	23.955; 0.0001
No	6 (18.2)	27 (81.8)	33	

* Pearson's chi-square

** Fishers exact test

Table 3 shows the logistic regression analysis of determinants of condom use at last sexual intercourse. These determinants included age 50-59 years (OR=3.34, 95% CI=1.21-9.25, p=0.020), lower education (OR=3.00, 95%CI=1.04-8.69, p=0.043), being married/have a partner (OR=3.25, 95%CI=1.11-9.52, p=0.031), partner's aware of respondents' HIV status (OR=13.00, 95%CI=4.25-39.80, p=0.0001) and stigma experience (OR=11.70, 95%CI=4.03-33.99, p=0.0001).

**Table 3:** Logistic regression analysis of selected factors associated with condom use at last sexual intercourse

Variable	OR	95%CI	p-value
Age group (years)			
50-59	3.34	1.21-9.25	0.020
≥60 (Ref)	1		
Level of education			
None/primary	3.00	1.04-8.69	0.043
Secondary/tertiary (Ref)	1		
Marital status			
Married/have a partner	3.25	1.11-9.52	0.031
Not married (Ref)	1		
Ethnicity			
Yoruba	0.25	0.08-0.75	0.013
Others (Ref)	1		
Partner aware of HIV status			
Yes	13.00	4.25-39.80	0.0001
No (Ref.)	1		
Stigma experience from partner			
Yes	11.70	4.03-33.99	0.0001
No (Ref.)	1		

Ref, reference; OR, odd ratio; CI, confidence interval

## Discussion

This study assessed the condom use and sexual behaviour of older adults living with HIV/AIDS at a treatment centre in southwest Nigeria. All the respondents were heterosexual in orientation while over two-fifth were sexually active. Although this finding is consistent with previous studies on sexual behaviour among HIV positive people, the fact that the respondents were aged 50 years and above shows that the perception that the older people were not sexually active was not totally correct. Publications among this population group support this finding^25^-^31^. Also, being HIV positive do not prevent engagement in sexual intercourse whether protected or high-risk sexual exposure. This study shows that the respondents' partners were aware of the respondents' HIV status with some respondents experiencing stigma. A previous study in this treatment center reported similar findings^9^. Although it is desirable for couples to be aware of each other HIV status, the mode of disclosure is very important to reduce the likelihood of stigma experience. It is necessary to put in place strategies that will encourage HIV status disclosure with reduced stigma experience. In addition, this will promote couple HIV counselling and testing among partners of people living with HIV/AIDS on care.

Furthermore, this study shows that some sexually active respondents did not use condom at last sexual exposure despite the provision of safer sex counselling sessions including male condoms in the centre. Previous studies in Nigeria and beyond have reported this finding^13^,^17^,^22^. Several reasons could be responsible for non-use of condom among these population. These could include non-disclosure of the respondent HIV status to partner, non-availability of condom at scene of sexual intercourse and refusal of either partners to use condom^15^,^17^,^18^. Also, some sexually active patients keep their sexual activities to themselves, hence did not benefit from the safer sex counseling session and male condom given at the centre. These high-risk behaviours have been shown to be responsible for the transmission of the virus to unsuspecting partners and others who have unprotected sex with them.

This study reported some significant factors associated with condom use at last sexual exposure among the study population. These factors included older age, being married or having a partner, having lower education, being unemployed, partner's awareness of respondents' HIV status and stigma experiencing. However, on further analysis, determinants of condom use at last sexual exposure included older age, lower education, partner's awareness of respondents' HIV status and stigma experience. The finding that higher proportion of respondents experiencing stigma fromtheir partners reported increased in condom use at last sexual intercourse could result out of fear of becoming infected with the virus. In order not to associate condom use with stigma experience and fear of infection, there is need for proper disclosure of HIV status by trained health care personnel. Also, proper HIV disclosure will encourage partner's participation in couple counselling and testing^9^,^19^,^29^.

## Limitation of the study

This study is limited by its cross-sectional design and the fact that some respondents could have given socially acceptable responses to some sensitive questions differing from their actual behaviour despite every effort made to explain the purpose of the study. Also, the respondents' partners were not interviewed hence could be using other forms of contraceptives that could affect their sexual practices and condom use.

## Conclusion

Sexually active older HIV-positive people engaged in high-risk sexual behaviour with inconsistent condom use at last sexual exposure indicating a need to strengthen the behavioural change communication strategy. It is necessary to put in place strategies that will encourage HIV status disclosure with reduced stigma experience which will promote HIV counseling and testing among partners of people living with HIV/AIDS on care. Policy makers need to refocus on the sexual health of the older people as they are not sexless as usually assumed. The older population should access HIV counseling and testing including comprehensive HIV care when found positive.
